# Diesel exhaust particles induce autophagy and citrullination in Normal Human Bronchial Epithelial cells

**DOI:** 10.1038/s41419-018-1111-y

**Published:** 2018-10-19

**Authors:** Tania Colasanti, Silvana Fiorito, Cristiano Alessandri, Annalucia Serafino, Federica Andreola, Cristiana Barbati, Francesca Morello, Michela Alfè, Gabriele Di Blasio, Valentina Gargiulo, Marta Vomero, Fabrizio Conti, Guido Valesini

**Affiliations:** 1 Rheumatology Unit, Department of Internal Medicine and Medical Specialties, Sapienza University of Rome, Rome, Italy; 2 Institute of Translational Pharmacology, CNR-Rome, Italy; 3 Istituto di Ricerche sulla Combustione (IRC), CNR-Naples, Italy; 4 Istituto Motori (IM), CNR-Naples, Italy

## Abstract

A variety of environmental agents has been found to influence the development of autoimmune diseases; in particular, the studies investigating the potential association of systemic autoimmune rheumatic diseases with environmental micro and nano-particulate matter are very few and contradictory. In this study, the role of diesel exhaust particles (DEPs), one of the most important components of environment particulate matter, emitted from Euro 4 and Euro 5 engines in altering the Normal Human Bronchial Epithelial (NHBE) cell biological activity was evaluated. NHBE cells were exposed in vitro to Euro 4 and Euro 5 particle carbon core, sampled upstream of the typical emission after-treatment systems (diesel oxidation catalyst and diesel particulate filter), whose surfaces have been washed from well-assessed harmful species, as polycyclic aromatic hydrocarbons (PAHs) to: (1) investigate their specific capacity to affect cell viability (flow cytometry); (2) stimulate the production of the pro-inflammatory cytokine IL-18 (Enzyme-Linked ImmunoSorbent Assay -ELISA-); (3) verify their specific ability to induce autophagy and elicit protein citrullination and peptidyl arginine deiminase (PAD) activity (confocal laser scanning microscopy, immunoprecipitation, Sodium Dodecyl Sulphate-PolyAcrylamide Gel Electrophoresis -SDS-PAGE- and Western blot, ELISA). In this study we demonstrated, for the first time, that both Euro 4 and Euro 5 carbon particles, deprived of PAHs possibly adsorbed on the soot surface, were able to: (1) significantly affect cell viability, inducing autophagy, apoptosis and necrosis; (2) stimulate the release of the pro-inflammatory cytokine IL-18; (3) elicit protein citrullination and PAD activity in NHBE cells. In particular, Euro 5 DEPs seem to have a more marked effect with respect to Euro 4 DEPs.

## Introduction

Diesel engines are one of the most important sources of anthropogenic particulate matter. The chemical composition of diesel exhaust particles (DEPs) consists of fine particles, <2.5 µm in diameter, and ultrafine particles (UFPs), <0.1 µm in diameter, with a center core of elemental carbon on which are absorbed organic and inorganic compounds, generally referred as soluble organic fraction (SOF), which includes partially burned fuel, lube oil residuals, tar-like species and polycyclic aromatic hydrocarbons (PAHs), most of them clearly harmful. These particles represent a big health concern, because they remain in the atmosphere for long periods, invade the indoor air environment, and can be breathed most deeply into the lungs. Harmful effects of DEPs on human health have been shown to include a higher risk for various diseases, particularly cancer, pulmonary and cardiovascular diseases^1–4^. Both in vitro and in vivo studies demonstrated the cytotoxicity of DEPs towards several cell lines and tissues. DEPs are efficiently internalized by different cell types, such as monocyte-derived macrophages, skin keratinocytes, lymphocytes and epithelial lung cells, in which induce pro-inflammatory molecule release, reactive oxygen species production, inhibition of anti-oxidative mechanisms and mortality^5–9^. Epidemiological studies on a great number of subjects living in proximity of roads with high density traffic have associated the exposure to UFPs to various diseases, including chronic obstructive pulmonary disease, pneumonia, heart attacks and autoimmune diseases^10–14^. To note, most studies focused on the effect of whole DEPs, without discriminating the effect of PAHs and other components from the effect of bare DEP surface.

Autoimmune diseases are complex disorders of unknown etiology. A variety of agents, such as viruses, hormones, drugs and pollutants, has been found to influence their development^15–18^. The studies investigating the potential association of systemic autoimmune rheumatic diseases (SARDs) with environment micro- and nano-particulate matter (PM) are few and contradictory. In mice models of collagen-induced arthritis, DEP exposure has been found to exacerbate the incidence and severity of the disease^19,20^. Recently, a significant association between PM < 2.5 µm levels and SARDs has been observed^21^. Epidemiological studies about the linkage between atmospheric pollution and rheumatoid arthritis (RA) showed that residential proximity to traffic was associated with an increased risk of this disease^22–24^. It is known that genetic (HLA-shared epitope) and environmental factors (i.e., cigarette smoke, air pollution) both might be the causes of the disease, even though their specific role is not well elucidated yet. A recent in vitro study demonstrated the pro-inflammatory effects of DEPs on scleroderma skin cells^25^, prompting a possible mechanism for PM-mediated effects. Environmental exposure to inhaled toxic substances has been shown to be able to induce citrullination in lung cells prior to any detectable onset of inflammatory responses, suggesting that this pathway may be important in linking environmental triggers to rheumatic disease risk^26,27^.

Citrullination is a post-translational modification catalysed by peptidylarginine deiminases (PADs), tissue specific enzymes involved in conversion of arginine to citrulline; it is a common feature of inflammation that results in protein conformation changes. As a consequence, citrullinated proteins can be recognized as 'non-self', and an autoimmune response can take place^28–31^.

Recent findings have underlined the key role also played by autophagy in the pathogenesis of some autoimmune diseases, such as RA and systemic lupus erythematosus (SLE)^32–34^. Autophagy represents the physiological mechanism by which aggregated proteins or dysfunctional cellular components are degraded and delivered to the lysosomes. An impaired control of autophagy contributes to the pathogenesis of many disorders, from neurodegenerative to inflammatory conditions, and may take part in the induction of autoimmunity^34^. Changes in the tissue environment may lead the local antigen presenting cells (APCs) to induce autophagy and with it citrullination, creating a substrate for autoreactivity. In this respect, autophagy in the APCs may represent the common feature of stress factors (smoking, joint injury, infection, etc.) that may drive the immune responses to citrullinated self-proteins^31^.

Because bronchial epithelial cells are directly exposed to DEPs in the air, these cells can be considered the first target of DEP cytotoxicity, inducing damage^7^ and, consequently, cellular loss of function. Since environmental pollutants can act as secondary factors in triggering the development and/or progression of many chronic and/or autoimmune diseases on a susceptible background, we aimed to investigate whether carbonaceous particles from Euro 4 and Euro 5 light duty diesel engine exhaust could affect in vitro normal human bronchial epithelial (NHBE) cell (a primary cell line isolated from the lower portion of the trachea, the carina and the bronchus) functionality, assessing the effects on the viability, in terms of autophagy, apoptosis and necrosis, and the ability to alter pro-inflammatory molecule production and to promote protein citrullination. In order to discriminate the specific effect of particle carbon core, this study has been conducted on DEPs deprived of SOF. Furthermore, the engine-out exhausts were sampled upstream of the typical emission after-treatment systems (diesel oxidation catalyst and diesel particulate filter), and are not representative of exhaust gases emitted from diesel engines working under strictly emission regulation.

## Materials and methods

### Cell culture

Frozen passage-1 stock of NHBE (Lonza, Slough, UK) cells was thawed and cultured in a T-25cm^2^ flask (Corning, NY, USA), pre-coated with a rat tail collagen Type 1 solution (30 μg/ml in phosphate-buffered saline -PBS-; BD Biosciences, New Jersey, USA), using Bronchial Epithelial cell Growth Medium (BEGM; Lonza), at 37 °C in an atmosphere of 5% CO_2_ and 95% relative humidity. The medium was changed every 2 days. When culture reached approximately 70% confluency, the cells were detached with 0.1% trypsin-ethylenediaminetetraacetic acid (EDTA; EuroClone, Pero, Milan, Italy) and were seeded at density of 3x10^5^ cells/cm^2^ on plates pre-coated with a 30 μg/ml collagen solution in PBS for 1 h, 37 °C. Cells were splitted at passages 2–4, using the ReagentPack subculture kit (Lonza), following suppliers instructions: BEGM was supplemented with hydrocortisone (0.5 mg/ml), insulin (5 mg/ml), transferrin (10 mg/ml), epinephrine (0.5 mg/ml), triiodothyronine (6.5 mg/ml), gentamycin (50 mg/ml), amphotericin-B (50 mg/ml), retinoic acid (0.1 ng/ml), and epidermal growth factor (0.5 ng/ml). At the passage 5, cells were treated with particle carbon core from diesel Euro 4 and Euro 5 engines; time to evaluate changes in the parameters analyzed was selected after preliminary experiments at 6, 16, 24 (Supplementary Fig. 2), and 48 h, at the concentrations of 3.3 and 6.6 μg/cm^2^. These concentrations were selected as the lowest concentration able to affect the examined parameters, without inducing excessive cytotoxicity, and in order to avoid the saturation of the response observed with higher doses (data not shown). The time course experiments showed that Euro 4 and Euro 5 DEPs should be used at 24 and 48 h of culture, depending on the parameter analyzed, to detect their maximum effect. As positive control for autophagy induction, cells underwent serum starvation for 16 h in 1:1 BEGM complete medium:BEGM without growth factors. Where indicated, in addition to DEPs, NHBE cells were cultured with lysosomal protease inhibitors E64d and Pepstatin A (PepA), both at 10 µg/ml (Sigma-Aldrich S.r.l., Milan, Italy), for 2 h before the end of the treatment.

### Particle collection and characterization

#### Experimental setup and particulate sampling

The DEP was sampled on a prototype single cylinder research engine, with a modern combustion system design derived from a Euro 5 compliant four-cylinder engine.

The operating points were performed using both Euro 4 and Euro 5 engine calibrations (derived from the real four-cylinder engine of equal unit displacement) that mainly differ in the exhaust-gas-recirculation rate, to ensure the value for practical application in the field of light duty engines. The tests were performed at fixed engine speed (2000 rpm) and load (5-bar brake mean effective pressure). More details about the experimental setup are reported in previous studies^25,35,36^. The engine-out exhausts were sampled as-emitted upstream of the typical emission after-treatment systems (diesel oxidation catalyst and diesel particulate filter).

#### DEP sampling and pretreatment

Total particulate was collected from the exhaust pipe by isokinetic sampling. The particulate matter collected on a Teflon filter was washed with dichloromethane (DCM), in order to remove condensable species, unburned fuel residuals and any interference of clearly harmful species possibly physiosorbed on the surface, as PAHs. The carbonaceous solid after DCM extraction was dried, weighted, and characterized. For the in vitro studies, the Euro 4 and Euro 5 carbon particles were sterilized by heating at 180 °C, at a temperature lower than that at the collection position (200 °C), in order to avoid affecting the particle properties. Then, the particles were washed three times in distilled water, suspended in PBS at a stock concentration of 1 mg/ml, and sonicated in a water bath at low intensity (1 on a scale of 3) for 48 h before the use, in order to obtain a better dispersion of the particles that tend to agglomerate.

#### DEP characterization

Fourier transform infrared spectra were recorded on a Nicolet iS10 spectrometer (ThermoFisher Scientific, Waltham, Massachusetts, USA), using the attenuated total reflection (ATR) method (ATR equipped with a germanium crystal). DEP spectral features and hydrodynamic diameter were evaluated in N-methylpyrrolidone (NMP) suspensions (DEP concentration was 10 μg/ml in NMP). The DEP hydrodynamic diameter was measured by using a Malvern Zetasizer Nano ZS instrument (Malvern Panalytical S.r.l., Lissone, Milan, Italy). The hydrodynamic diameter of single particles has been evaluated not only in NMP (without the occurrence of aggregation phenomena^35,36^), but also in PBS and in supplemented cell culture medium (where aggregation phenomena are expected due to the hydrophobic nature of the DEPs). Hence, Dynamic Light Scattering (DLS) measurements were performed in different media (NMP, PBS and supplemented cell culture medium), in order to assess the occurrence of aggregation phenomena. In DLS measurements, DEP concentration was 10 μg/ml. Ultraviolet (UV)-Visible (Vis) spectra of DEPs were acquired on HP 8453 diode array spectrophotometer. Being the DEP molecular mass unknown, the absorption coefficients have been expressed on a mass basis (m^2^/g). The surface characteristics of the particulates were probed by Electron Energy Loss Spectroscopy (EELS) and ATR-InfraRed (ATR-IR) spectroscopy. Carbon- K-ionization edge measurements were performed using EELS. More details are reported in the study of Mastrofrancesco *et al*.^25^.

### Confocal laser scanning microscopy (CLSM)

Autophagy induction was assessed by analyzing the redistribution at cytoplasmic vacuoles of the autophagy membrane marker LC3B (hereafter referred as LC3). Cells were grown on cover-slips and treated with Euro 4 or Euro 5 diesel particle carbon core for 48 h. Treated cells and untreated control were fixed with 2% paraformaldehyde in PBS (Sigma-Aldrich) and permeabilized with 0.2% Triton X-100 (Sigma-Aldrich), and then subjected to immunofluorescent staining, using the rabbit polyclonal antibody against LC3 (ABGENT, San Diego, CA, USA; working dilution 1:100). The primary antibody was detected with Alexa Fluor 488-conjugated anti-rabbit IgG (Molecular Probes, Oregon, USA) and samples were observed by LEICA TCS SP5 CLSM (Leica Instruments, Mannheim, Germany). The percentage of autophagic LC3-positive (LC3^+^) cells (>3 punctate staining sites per cell) was obtained analyzing in a blinded fashion a minimum of 200 cells/sample by CLSM. Data were from three experiments (*N* = 3) and presented as means ± SD.

### Sodium dodecyl sulphate-polyacrylamide gel electrophoresis (SDS-PAGE) and Western blot

NHBE cells were lysed in lysis buffer (100 mM Tris-HCl, pH 8; 150 mM NaCl; 1% Triton X-100; 1 mM MgCl_2_; 25 mM Na_3_VO_4_), in the presence of Complete protease inhibitor mixture (Roche Diagnostics, Deutschland GmbH, Mannheim, Germany). Protein content was determined by the Bradford assay (BioRad Laboratories, Richmond, CA, USA). NHBE cells lysates (30 µg) were loaded onto a 15% SDS-PAGE in denaturing conditions and, after electrophoresis, proteins were transferred onto polyvinylidene difluoride membrane (Amersham Hybond-ECL, GE Healthcare Europe, Munich, Germany) by means of a Trans-Blot transfer cell (BioRad Laboratories). All the membranes were cropped at the molecular weight of interest, to enhance the antibody yield. For citrullinated protein detection, to improve protein retention because these proteins tend to easily detach from blotted membranes, the method described by Colasanti *et al*.^37^ was used. The membranes were then blocked in 5% skim milk in Tris-buffered saline containing 0.1% Tween 20 (TBS-T) for 1 h at room temperature, rinsed, and incubated with the intended antibodies (Abs). As primary Abs, rabbit IgG anti-LC3B (LC3B D11 XP Rabbit mAb, Cell Signaling Technology, Leiden, The Netherlands) and anti-p62/SQSTM1 (hereafter referred as p62; Sigma-Aldrich) Abs were used at a dilution of 1:1000 in TBS-T with 5% bovine serum albumin (BSA); F95 mouse IgM anti-citrullinated proteins (Anti-peptidyl-citrulline, clone F95; Merck Millipore, Vimodrone, Milan, Italy) were used at a dilution of 1:500 in TBS-T with 5% skim milk.

Immunoprecipitate samples (20 μg) were equally subjected to 10% SDS-PAGE in denaturing conditions and immunoblotting, with rabbit anti-vimentin as control (Vimentin D21H3 XP Rabbit mAb, Cell Signaling Technology; dilution 1:1000 in TBS-T containing 5% BSA) and F95 mouse anti-citrullinated protein (used as previously indicated) Abs.

Excess primary antibody was removed by washing the membranes in TBS-T. The membranes were then incubated with horseradish peroxidase (HRP)-conjugate goat anti-rabbit IgG (BioRad Laboratories) or mouse anti-human IgM (Jackson ImmunoResearch Laboratories Inc., West Grove, PA, USA) Abs, 1:3000 in TBS-T with 5% skim milk. The reaction was developed using the chemiluminescent HRP detection reagent Luminata Forte (Merck Millipore). A rabbit IgG anti-β-actin Ab (Sigma-Aldrich) was used as protein content control. Quantification of protein expression was performed by densitometry analysis of the autoradiograms (GS-700 Imaging Densitometer, BioRad Laboratories). The results shown are mean ± SD of nine experiments (*N* = 9).

### Immunoprecipitation

Cell-free lysates from NHBE cells, untreated or exposed to DEPs for 48 h, were immunoprecipitated with rabbit IgG anti-vimentin (5 μg) or with irrelevant IgG (Irr. IgG), as a negative control (5 μg). In brief, following manufacturer’s instructions for ThermoFisher Scientific PierceDirect IP Kit (Pierce Biotechnology, Rockford, USA), cells were lysed in lysis buffer as previously described and, to preclear nonspecific binding, cell-free lysates were mixed with Control Agarose Resin slurry and stirred in a rotary shaker for 1 h at 4 °C. After centrifugation (1000x*g* for 1 min), vimentin was immunoprecipitated from the precleared samples, as indicated in the manifacturer’s instructions.

### Flow cytometry

Apoptosis and necrosis of NHBE cells after 24 and 48 h by DEP exposure were quantified using a fluorescein isothiocyanate-conjugate Annexin V (AV) and phycoerythrin-conjugate propidium iodide (PI) apoptosis detection kit, according to the recommendations of the manufacturer (Marine Biological Laboratory, Woods Hole, MA, USA). Reported data were referred to AV-positive (AV^+^) and PI-positive (PI^+^) cells. Acquisition was performed on a FACSCalibur cytometer (BD Biosciences), and 20,000 events/sample were run. Data were analyzed using the CellQuest software (BD Biosciences). The results represent the mean ± SD of nine experiments (*N* = 9).

### Peptidylarginine deiminase (PAD) activity

NHBE cells untreated or treated for 48 h with 3.3 and 6.6 μg/cm^2^ of DEPs from diesel Euro 4 and Euro 5 engines, in the presence or in the absence of the calcium chelator EDTA, were lysed as previously described. Equal amounts of whole cellular extracts were analyzed using an Antibody Based Assay for PAD activity (ModiQuest Research, AC Oss, The Netherlands), a solid enzyme-linked immunosorbent assay (ELISA), following the manufacturer’s instructions. The optical density at 490 nm was directly correlated to the control enzyme activity present in the test, resulting in a quantitation of the PAD enzymatic activity. All the lysates were run in quadruplicate in three different assays (*N* = 3 and, within each test, all the samples were tested in 4 distinct wells) and the data shown represent the mean ± SD.

### IL-18 production

Supernatants were collected from NHBE cells untreated or incubated with Euro 4 and Euro 5 DEPs, at concentration of 3.3 and 6.6 μg/cm^2^. A commercially available ELISA kit was used to determine the levels of IL-18 (Human IL-18 ELISA Kit; MBL, Woburn, MA, USA) in the culture supernatants, according to the manufacturer’s instructions. The incubation time of 24 h was preliminarily selected, analyzing the release of IL-18 after 6, 16 (data not shown), and 24 h, and the results were virtually the same.

The intra-assay variation ranged from 5–11% and the inter-assay variation ranged from 5–10%. The limit of detection (sensitivity of the assay) was 12.5 pg/ml. All the supernatants were run in quadruplicate in three different assays (*N* = 3 and, within each test, all the samples were tested in 4 distinct wells) and data were reported as mean ± SD.

### Statistical analysis

All data were expressed as mean ± SD. Results were analyzed using GraphPad Prism v6 (GraphPad Inc., San Diego, CA, USA). The Mann Whitney unpaired test or the Student’s T test were used to compare quantitative variables in different treatment groups. Significant *P* values were indicated as <0.05.

## Results

### Particle characterization

A detailed description of the chemico-physical and structural properties of Euro 4 and Euro 5 DEPs was given in previous studies^25,35,36^. For sake of clarity, a summary of the main results is reported in the following and in Supplementary material.

It was estimated that, among the species removed by DCM extraction, SOF accounts for 8–10 wt % of the total particulate mass in both engine configurations (Euro 4 and 5).

Both particulates showed a similar micro and nano-texture, consisting of irregularly shaped compact aggregates of almost spherical primary particles (diameter 10–20 nm; Supplementary Fig. 1a).

The Electron energy Loss Near-Edge Structures of the carbon-K-ionization edge were deduced from the EELS spectra (Supplementary Fig. 1b). The predominant binding properties were similar in both DEPs and agree with EELS data reported for other diesel particulate samples^35,36,38,39^.

In ATR-IR spectra, both DEPs appeared quite similar and no significant differences were detectable. The broad shape of the spectra was typical of a complex carbon network^40^ (Supplementary Fig. 1b).

The UV-Vis DEP spectra (Supplementary Fig. 1c) exhibited a broad shape degrading from UV toward visible region, typical of complex carbon-based materials produced in combustion processes^40,41^. The spectral shape appeared quite similar for both samples, but the specific absorption values were different; in particular, they were higher for Euro 5 than Euro 4 DEPs, indicating that the graphitization degree was slightly more pronounced in the Euro 5 DEPs, in accordance with EELS data. Overall, this specific absorptions were similar to those of carbons with a high graphitization degree and a good level of structuration^40,41^. The adsorption values of Euro 5 DEPs were in accordance with data reported by Gargiulo *et al*.^36^, confirming the reproducibility of the combustion system and the methodology of total particulate sampling.

Firstly, DLS was performed in NMP suspension, in order to estimate the hydrodynamic diameter of the particles without the occurrence of aggregation phenomena^41^. The hydrodynamic diameters of Euro 5 appeared slightly larger (115 ± 5 nm) than Euro 4 (95 ± 5 nm) DEPs. The analyzed samples appeared quite monodispersed (in both cases, the polydispersity index -PI- was less than 0.3). When suspended in PBS, the hydrodynamic diameters of both DEPs appeared quite larger, compared to those evaluated in NMP (Euro 5 DEPs: 2750 ± 80 nm; Euro 4 DEPs: 1700 ± 100 nm, PI = 0.9 in both cases) indicating, as expected, the occurrence of aggregation phenomena. When mixed with the supplemented cell culture medium, the particle size distribution appeared broader and polydisperse, with diameters ranging from 570 nm to 4500 nm. Due to the instability of the suspension, a reliable evaluation of the particle size distribution was not possible.

### DEPs are able to induce autophagy in NHBE cells

**Fig. 1:**
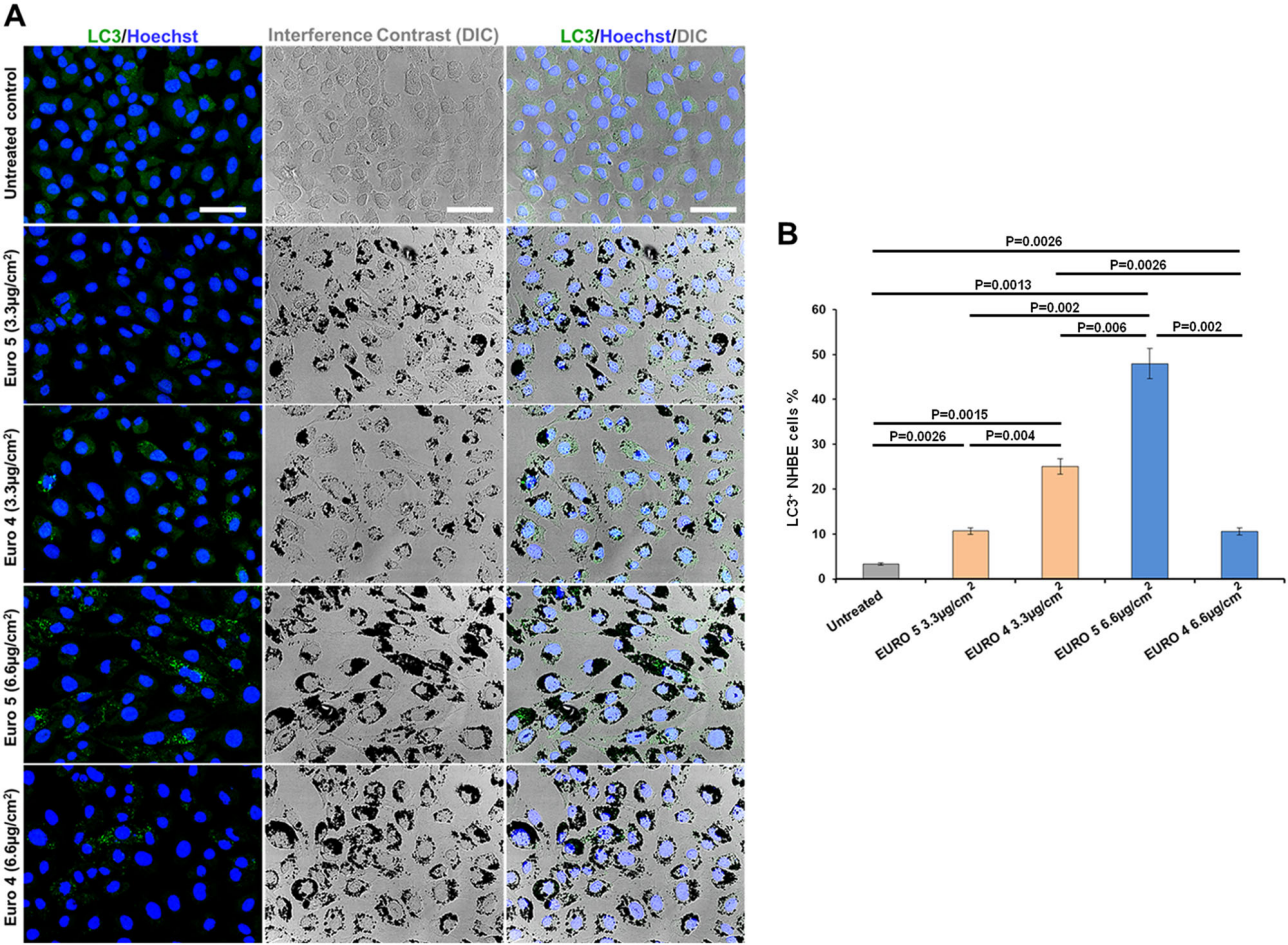
Effects of DEPs on autophagy: induction of autophagic vacuoles in Normal Human Bronchial Epithelial (NHBE) cells by Euro 5 and Euro 4 carbon particles, before any after-treatment. **a** Confocal microscopy of autophagy induction, evaluated by LC3 immunostaining after 48 h of treatment with the diesel particle carbon core, before any after-treatment. Single and merged images of differential interference contrast (DIC), LC3 immunofluorescent staining (green hue) and nuclei staining with Hoechst (blue hue) are shown. Bars 50 µm. **b** Quantitative assessment of autophagic LC3-positive (LC3^+^) cells, performed as described in Materials and Methods section; results are reported as percentage of LC3^+^ cells. For 3.3 μg/cm^2^: *P* = 0.0026 Euro 5 *vs* untreated; *P* = 0.0015 Euro 4 *vs* untreated; *P* = 0.004 Euro 5 *vs* Euro 4. For 6.6 μg/cm^2^: *P* = 0.0013 Euro 5 *vs* untreated; *P* = 0.0026 Euro 4 *vs* untreated; *P* = 0.002 Euro 5 *vs* Euro 4. For a dose-depending comparison: *P* = 0.002 Euro 5 3.3 *vs* 6.6 μg/cm^2^; *P* = 0.0026 Euro 4 3.3 *vs* 6.6 μg/cm^2^. *P* = 0.006 for Euro 4 3.3 μg/cm^2^ *vs* Euro 5 6.6 μg/cm^2^. Results are represented as the mean ± SD of three experiments (*N* = 3)

We examined the response of NHBE cells to Euro 4 and Euro 5 DEPs, in terms of susceptibility to autophagy induction. For this purpose we firstly analyzed, by CLSM, the redistribution at cytoplasmic vacuoles of the autophagy marker LC3 in NHBE cells. In Fig. 1a, b, it can be seen an increase in the percentage of autophagic LC3^+^ cells, after treatment with Euro 4 and Euro 5 DEPs for 48 h.

LC3 is considered critical for autophagy; it is firstly cleaved at the carboxy terminus to yield the cytosolic form LC3-I. Subsequently, LC3-I is converted to LC3-II through lipidation and, in this form, it becomes associated with autophagic vesicles. For this reason, LC3-II is used as a marker of autophagy^32,33^.

**Fig. 2:**
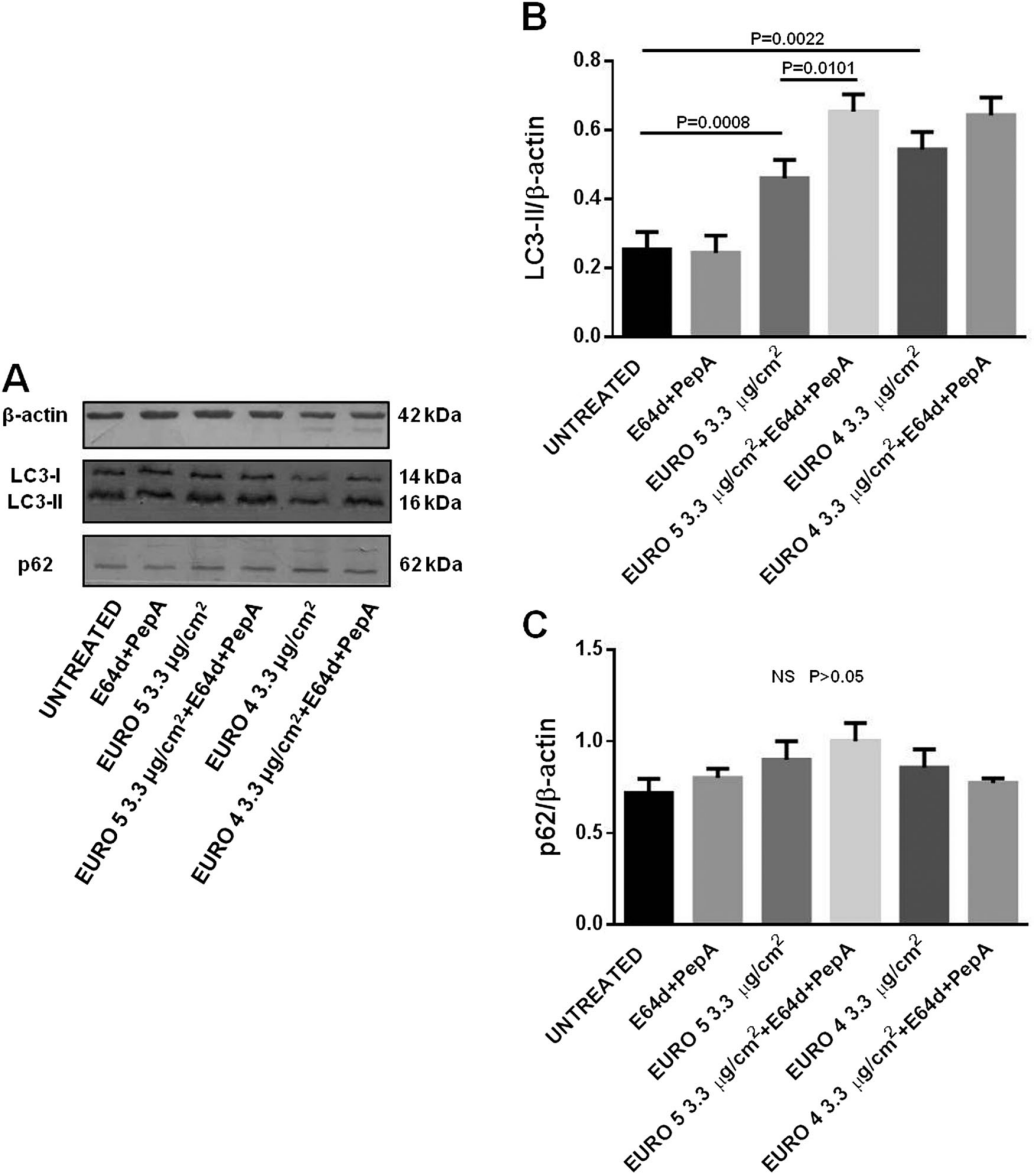
Effects of Euro 5 and Euro 4 DEPs, before any after-treatment, at 3.3 μg/cm^2^ on autophagy: evaluation of LC3-II and p62 levels as autophagic markers in Normal Human Bronchial Epithelial (NHBE) cells. **a** Western blot analysis of LC3-II and p62 levels in lysates from NHBE cells (i) untreated and cultured for 48 h with (ii) lysosomal protease inhibitors E64d and Pepstatin A (PepA), (iii) Euro 5, (iv) Euro 5 + E64d + PepA, (v) Euro 4, (vi) Euro 4 + E64d + PepA. Blot shown is representative of nine experiments (*N* = 9). **b** Densitometry analysis of LC3-II levels relative to β-actin (mean ± SD). *P* = 0.0008 for Euro 5 *vs* untreated; *P* = 0.0101 for Euro 5 *vs* Euro 5 + E64d + PepA; *P* = 0.0022 for Euro 4 *vs* untreated. **c** Densitometry analysis of p62 levels relative to β-actin (mean ± SD). No significant (NS, *P* > 0.05) differences in p62 levels were found

**Fig. 3:**
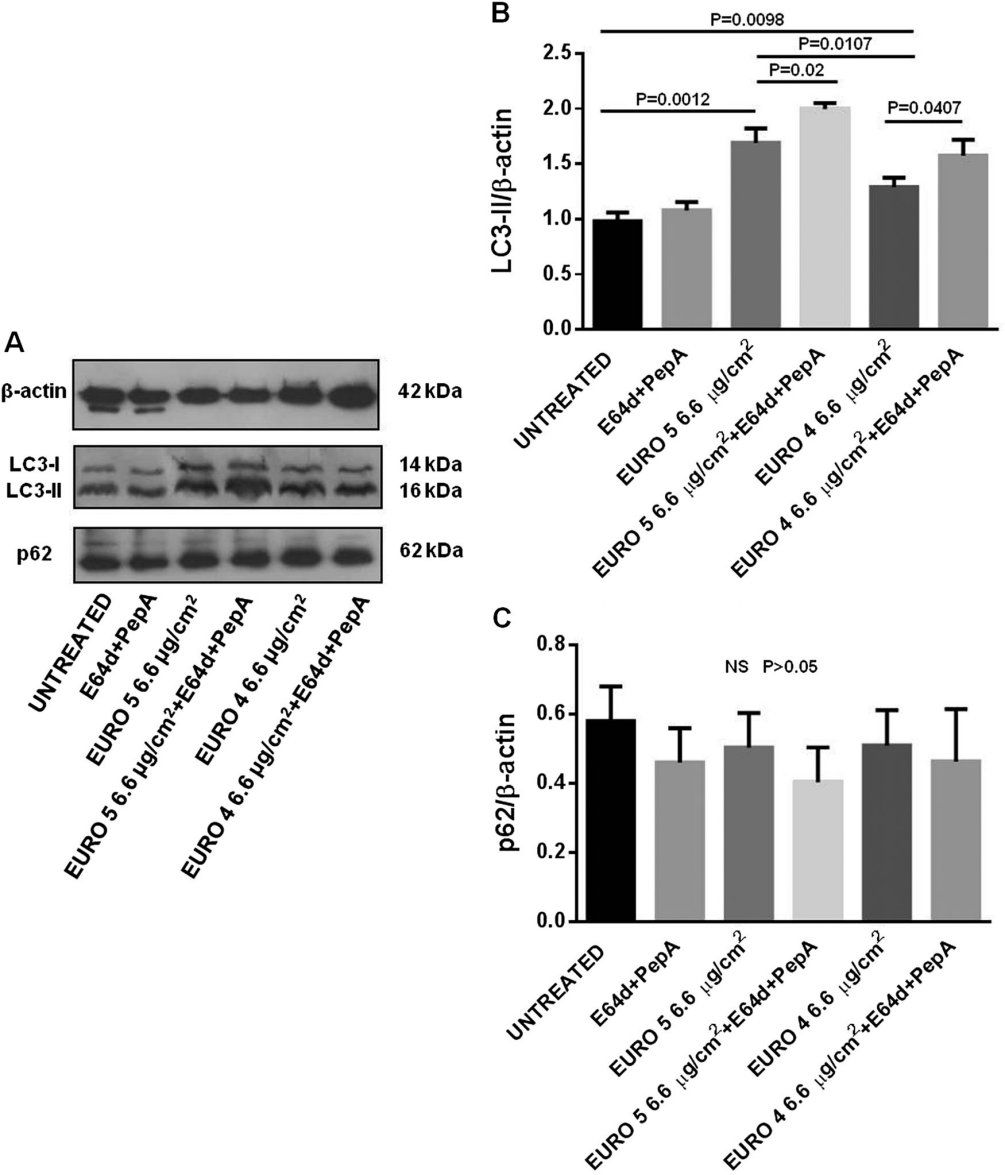
Effects of Euro 5 and Euro 4 DEPs, before any after-treatment, at 6.6 μg/cm^2^ on autophagy: evaluation of LC3-II and p62 levels as autophagic markers in Normal Human Bronchial Epithelial (NHBE) cells. **a** Western blot analysis of LC3-II and p62 levels in lysates from NHBE cells (i) untreated and cultured for 48 h with (ii) E64d + PepA, (iii) Euro 5, (iv) Euro 5 + E64d + PepA, (v) Euro 4, (vi) Euro 4 + E64d + PepA. Blot shown is representative of nine experiments (*N* = 9). **b** Densitometry analysis of LC3-II levels relative to β-actin (mean ± SD). *P* = 0.0012 for Euro 5 *vs* untreated; *P* = 0.02 for Euro 5 *vs* Euro 5 + E64d + PepA; *P* = 0.0098 for Euro 4 *vs* untreated; *P* = 0.0407 for Euro 4 *vs* Euro 4 + E64d + PepA; *P* = 0.0107 for Euro 5 *vs* Euro 4. **c** Densitometry analysis of p62 levels relative to β-actin (mean ± SD). No significant (NS, *P* > 0.05) differences in p62 levels were found

Successively, we analyzed the differences in the autophagic flux by Western blot, evaluating the LC3-II levels, in presence/absence of the specific lysosomal protease inhibitors E64d and PepA, in lysates from NHBE cells treated with DEPs. After 24 h of both Euro 4 and Euro 5 DEP exposure, an accumulation of LC3-II started to occur (Supplementary Figure 2c), but a more significant increase was observed after 48 h. Indeed, as shown in Fig. 2a, b, a statistically significant increase of LC3-II level was observed in NHBE cells, in presence of Euro 5 and Euro 4 particles at the concentration of 3.3 μg/cm^2^, with an effect due to Euro 4 > Euro 5; only cells treated with Euro 5, in presence of E64d and PepA, exhibited a significant increase of LC3-II level, with respect to their specific control sample without lysosomal inhibitors. At the concentration of 6.6 μg/cm^2^ (Fig. 3a, b), considerable significant changes in LC3-II level were induced in cells exposed to both DEPs, the presence of lysosomal inhibitors significantly enhanced the autophagic flux, and the effect of Euro 5 DEPs was more evident. So, NHBE cells exposed to both DEPs showed higher levels of LC3-II.

Lysosomal protease inhibitors were used in order to understand if LC3-II accumulation was due to an increased autophagosome formation or an impaired lysosomal function. NHBE cells exposed to DEPs showed an increase of LC3-II levels, with further accumulation following the addition of E64d/PepA, this being congruent with an induction of LC3-II degradation at the autophagolysosomal level.

In order to investigate the autophagic flux more accurately, we measured the levels of p62. Since this protein may serve to associate ubiquitinated proteins to the autophagic machinery to allow their degradation in the lysosome, p62 may be used as an autophagic flux marker^42^. As indicated in Figs. 2a, c, and 3a, c, no significant differences in p62 amount were found, as compared to the p62 basal level, in NHBE cells exposed for 48 h to Euro 5 and Euro 4 particles at both the experimental concentrations observed, even in the presence of E64d and PepA.

In order to trigger a metabolic impairment, leading to a starvation-induced autophagy, NHBE cells were also cultured for 16 h in culture medium without growth factors (data not shown).

Altogether, the data obtained by the analysis of the autophagic markers LC3-II (significantly increased, also in presence of lysosomal protease inhibitors, with a slightly more significant effect at the concentration of 6.6 μg/cm^2^) and p62 (not significantly changed in all the experimental conditions) indicate that the differences in LC3-II level correspond to an effective increase of the autophagic flux due to DEPs exposure.

### DEPs affect cell viability inducing apoptosis and necrosis in NHBE cells

**Fig. 4:**
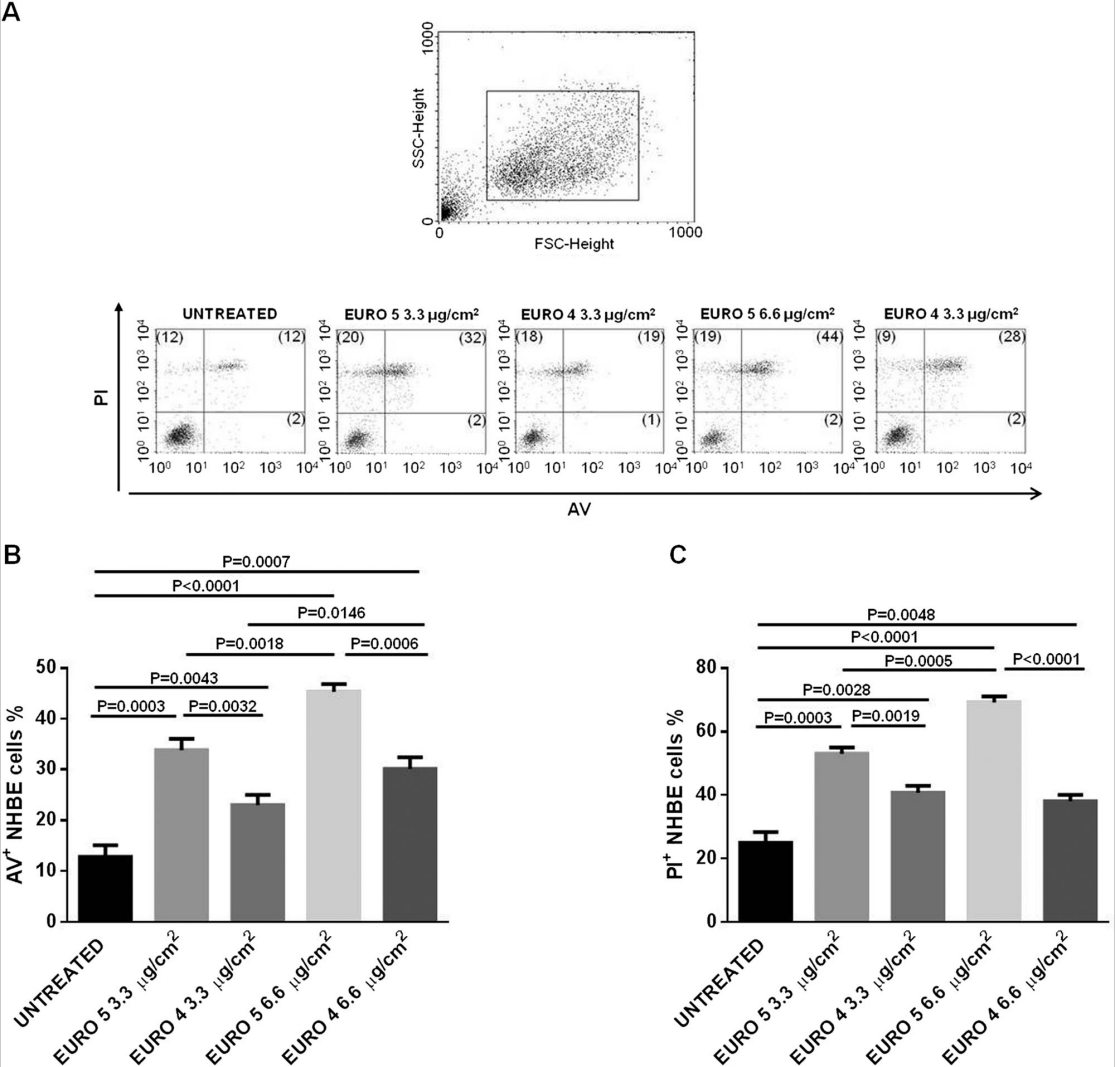
Effect of Euro 5 and Euro 4 DEPs, before any after-treatment, on cell viability: evaluation of apoptosis and necrosis in Normal Human Bronchial Epithelial (NHBE) cells. **a** Morphological characterization and analysis of apoptosis and necrosis by flow cytometry in NHBE cells untreated and treated for 24 h with Euro 5 and Euro 4 DEPs, before any after-treatment. Results shown are representative of three experiments in triplicate (*N* = 9). Numbers in upper and bottom right quadrants of each plot refer to Annexin V positive (AV^+^) cells, while numbers in the upper quadrants (left and right) represent Propidium Iodide positive (PI^+^) cells. **b** DEPs induce apoptosis in NHBE cells, reported as the percentage of AV^+^ cells. For 3.3 μg/cm^2^: *P* = 0.0003 for Euro 5 *vs* untreated; *P* = 0.0043 for Euro 4 *vs* untreated; *P* = 0.0032 for Euro 5 *vs* Euro 4. For 6.6 μg/cm^2^: *P* < 0.0001 for Euro 5 *vs* untreated; *P* = 0.0007 for Euro 4 *vs* untreated; *P* = 0.0006 for Euro 5 *vs* Euro 4. For a dose-depending comparison: *P* = 0.0018 for Euro 5 3.3 *vs* 6.6 μg/cm^2^; *P* = 0.0146 for Euro 4 3.3 *vs* 6.6 μg/cm^2^. **c** DEPs increase the percentage of necrotic (PI^+^) NHBE cells. For 3.3 μg/cm^2^: *P* = 0.0003 for Euro 5 *vs* untreated; *P* = 0.0028 for Euro 4 *vs* untreated; *P* = 0.0019 for Euro 5 *vs* Euro 4. For 6.6 μg/cm^2^: *P* < 0.0001 for Euro 5 *vs* untreated; *P* = 0.0048 for Euro 4 *vs* untreated; *P* < 0.0001 for Euro 5 *vs* Euro 4. For a dose-depending comparison: *P* = 0.0005 for Euro 5 3.3 *vs* 6.6 μg/cm^2^; *P* > 0.05 for Euro 4 3.3 *vs* 6.6 μg/cm^2^

In order to verify any differences in terms of apoptosis and necrosis levels in response to DEP treatment, we used a dual staining with AV and PI. NHBE cells were treated for 24 and 48 h with Euro 4 and Euro 5 particles at the concentrations of 3.3 and 6.6 μg/cm^2^. In Fig. 4a, b, a significant increase of apoptotic cells in samples exposed to both DEPs at 24 h was shown. It’s remarkable the Euro 4 particle ability to induce NHBE cell apoptosis at a lower extent and in a dose-dependent manner, with respect to Euro 5.

DEP exposure elicited necrosis in NHBE cells (Fig. 4a, c) and, as well as for apoptosis, the greater effect was due to NHBE cell treatment with Euro 5 DEPs, which induced a dose-dependent increase of necrotic cells.

After 48 h of DEP exposure, only Euro 4 particles at 6.6 μg/cm^2^ seem to exert their effect, increasing AV^+^ cells, as well as the percentage of PI^+^ cells, at both the doses used (Supplementary Fig. 3a, b).

### DEPs induce protein citrullination in NHBE cells

To investigate the potential effect of Euro 4 and Euro 5 DEPs on protein citrullination, NHBE cells were exposed for 48 h to 3.3 and 6.6 μg/cm^2^ of DEPs.

**Fig. 5:**
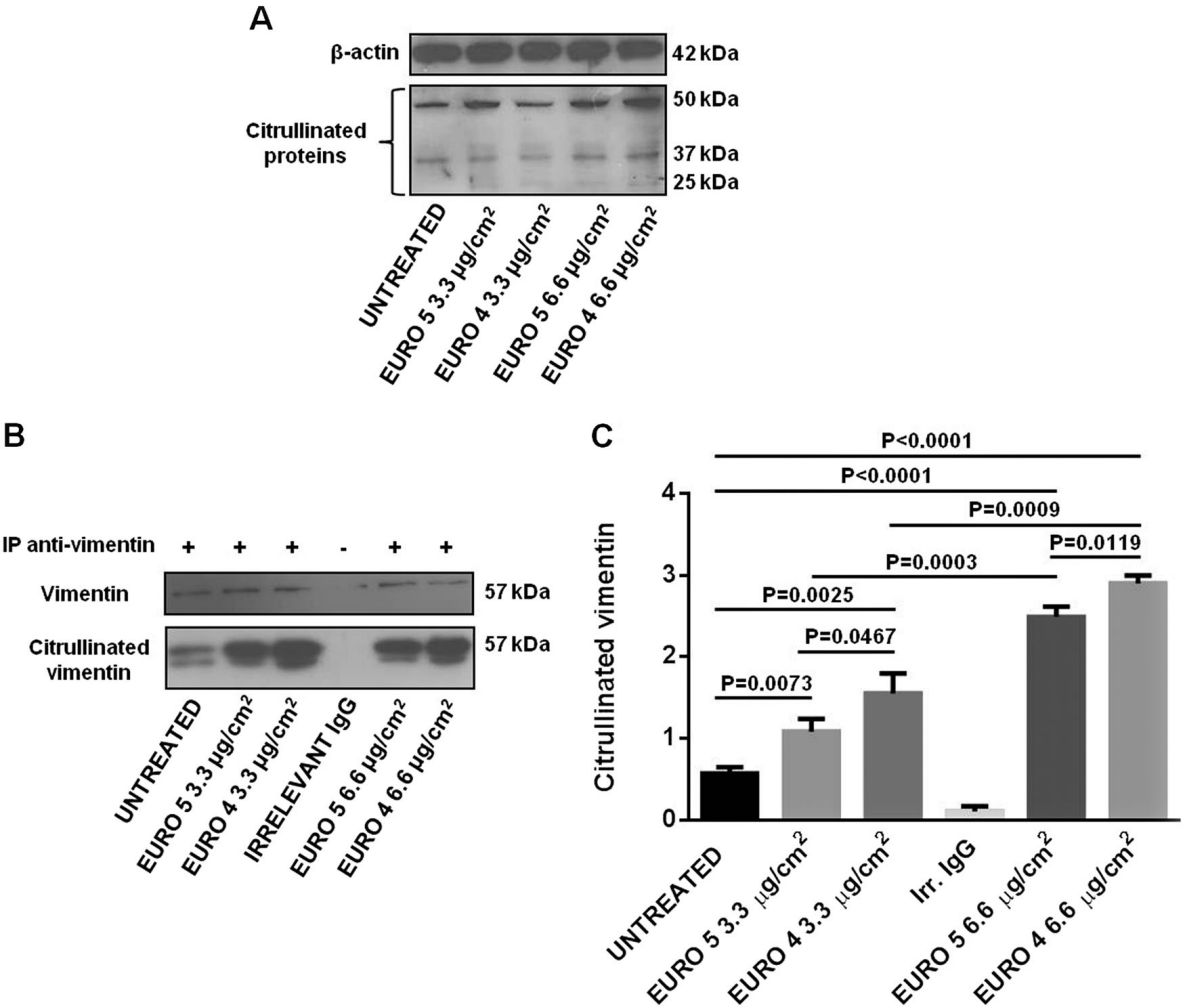
Effect of Euro 5 and Euro 4 DEPs, before any after-treatment, in enhancing citrullinated protein levels in Normal Human Bronchial Epithelial (NHBE) cells. **a** Western blot analysis of citrullinated protein levels in lysates from NHBE cells (i) untreated and cultured for 48 h with (ii) Euro 5 3.3 μg/cm^2^, (iii) Euro 4 3.3 μg/cm^2^, (iv) Euro 5 6.6 μg/cm^2^, (v) Euro 4 6.6 μg/cm^2^. Blots shown are representative of three experiments (*N* = 3). **b** Cells were lysed and vimentin was immunoprecipitated from lysates. An appropriate IgG isotypic control (irrelevant IgG, Irr. IgG) was employed to verify that immunoprecipitation was correctly performed. Blots shown are representative of nine experiments (*N* = 9). **c** Densitometry analysis of citrullinated vimentin levels relative to vimentin (mean ± SD). For 3.3 μg/cm^2^: *P* = 0.0073 Euro 5 *vs* untreated; *P* = 0.0025 Euro 4 *vs* untreated; *P* = 0.0467 Euro 5 *vs* Euro 4. For 6.6 μg/cm^2^: *P* < 0.0001 Euro 5 and Euro 4 *vs* untreated; *P* = 0.0119 Euro 5 *vs* Euro 4. For a dose-depending comparison: *P* = 0.0003 Euro 5 3.3 *vs* 6.6 μg/cm^2^; *P* = 0.0009 Euro 4 3.3 *vs* 6.6 μg/cm^2^

The NHBE cell lysates were probed with F95 Ab. This method allowed to observe the citrullinated protein bands between 43 and 65 kDa. The induction of protein citrullination by Euro 4 and Euro 5 particles was monitored by the detection of these bands (Fig. 5a).

Then, lysates were tested for the presence of citrullinated vimentin by immunoprecipitation, using an anti-vimentin Ab, and by subsequent immunoblotting with F95 Ab (Fig. 5b). As control, immunoprecipitates were checked for the vimentin content. At both DEP concentrations, the densitometric assessment of citrullinated vimentin showed a significant increase in protein citrullination in NHBE treated cells (Fig. 5c). Euro 4 DEPs were able to cause a significant increase in protein citrullination level, in a dose-dependent manner and more dramatically, with respect to Euro 5 DEPs.

### DEPs increase PAD enzymatic activity in NHBE cells

**Fig. 6:**
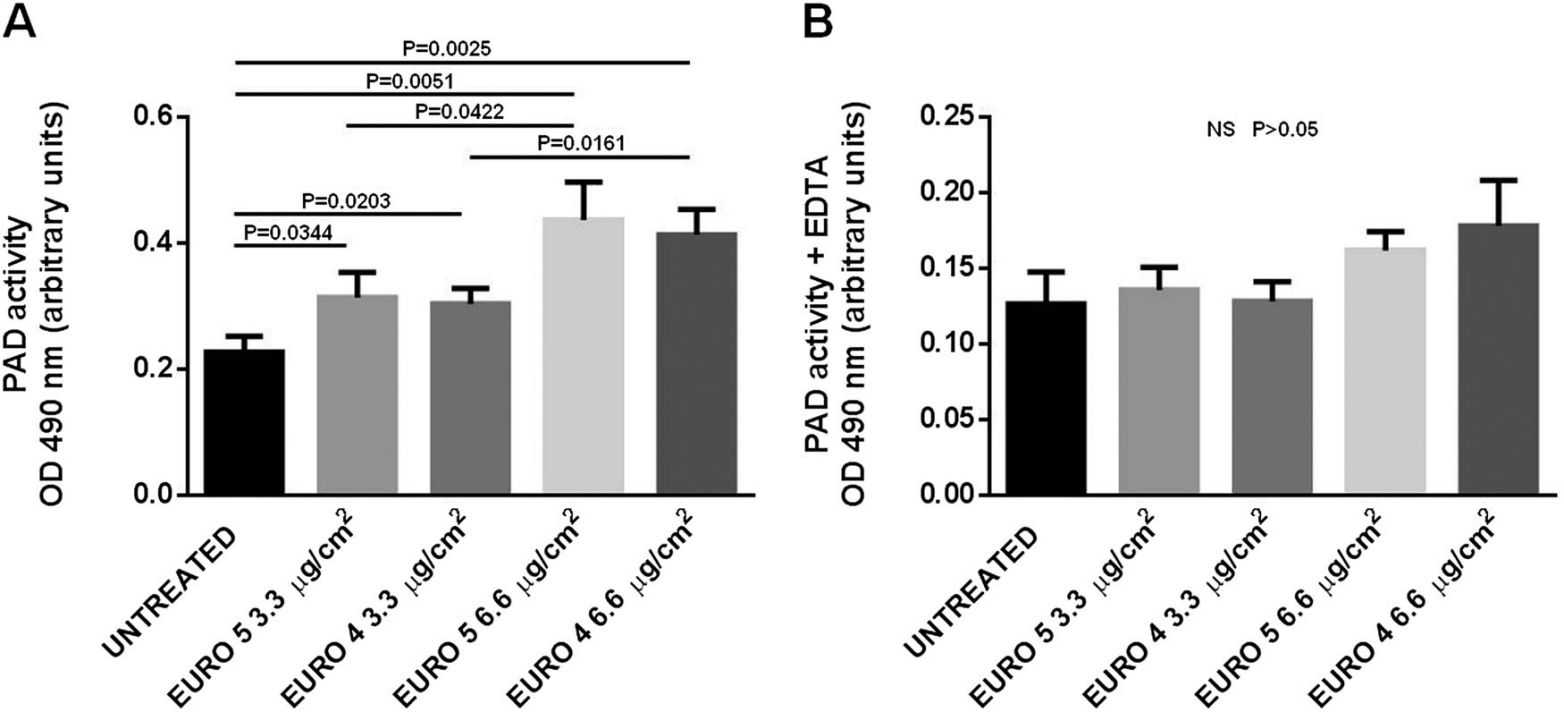
Effect of Euro 5 and Euro 4 DEPs, before any after-treatment, on PAD enzymatic activity induction. PAD activity was assessed in lysates **a** from NHBE cells (i) untreated and cultured for 48 h with (ii) Euro 5 3.3 μg/cm^2^, (iii) Euro 4 3.3 μg/cm^2^, (iv) Euro 5 6.6 μg/cm^2^, (v) Euro 4 6.6 μg/cm^2^. Quadruplicate analyses (replicates) were conducted for all the lysates in three different assays (*N* = 3) and results obtained are shown as mean ± SD. For 3.3 μg/cm^2^: *P* = 0.0344 Euro 5 *vs* untreated; *P* = 0.0203 Euro 4 *vs* untreated. For 6.6 μg/cm^2^: *P* = 0.0051 Euro 5 *vs* untreated; *P* = 0.0025 Euro 4 *vs* untreated. For a dose-depending comparison: *P* = 0.0422 Euro 5 3.3 *vs* 6.6 μg/cm^2^; *P* = 0.0161 Euro 4 3.3 *vs* 6.6 μg/cm^2^. **b** Since Ca^2^ + is required for PAD activity, the background of the assay was set up by analyzing each lysate in the presence of the chelating agent EDTA. No increase in PAD activity was observed, following treatment with Euro 4 and Euro 5 DEPs (NS, *P* > 0.05 for all the experimental conditions). Quadruplicate analyses (replicates) were conducted for all the lysates in three different assays (*N* = 3) and results obtained are shown as mean ± SD

To completely evaluate the protein citrullination process in NHBE cells exposed to DEPs for 48 h, we also assessed PAD enzymatic activity. As shown in Fig. 6a, Euro 4 and Euro 5 particles at 3.3 μg/cm^2^ induced a significant increase in PAD activity. In the same way at 6.6 μg/cm^2^, both DEPs were able to induce an enzymatic activity enhancement, but this DEP concentration seemed to exert a stronger effect.

Since Ca^2+^ is required for PAD activity, the background of the assay was set up by analyzing each sample in the presence of the chelating agent EDTA; as expected, no increase in PAD activity was observed, following treatment with Euro 4 and Euro 5 DEPs (Fig. 6b).

### DEPs are able to induce IL-18 production in NHBE cell culture supernatants

Because of the pro-inflammatory role played by IL-18, the production/release of this cytokine in the culture medium of NHBE cells exposed to Euro 4 and Euro 5 DEPs for 24 h was evaluated by ELISA.

**Fig. 7:**
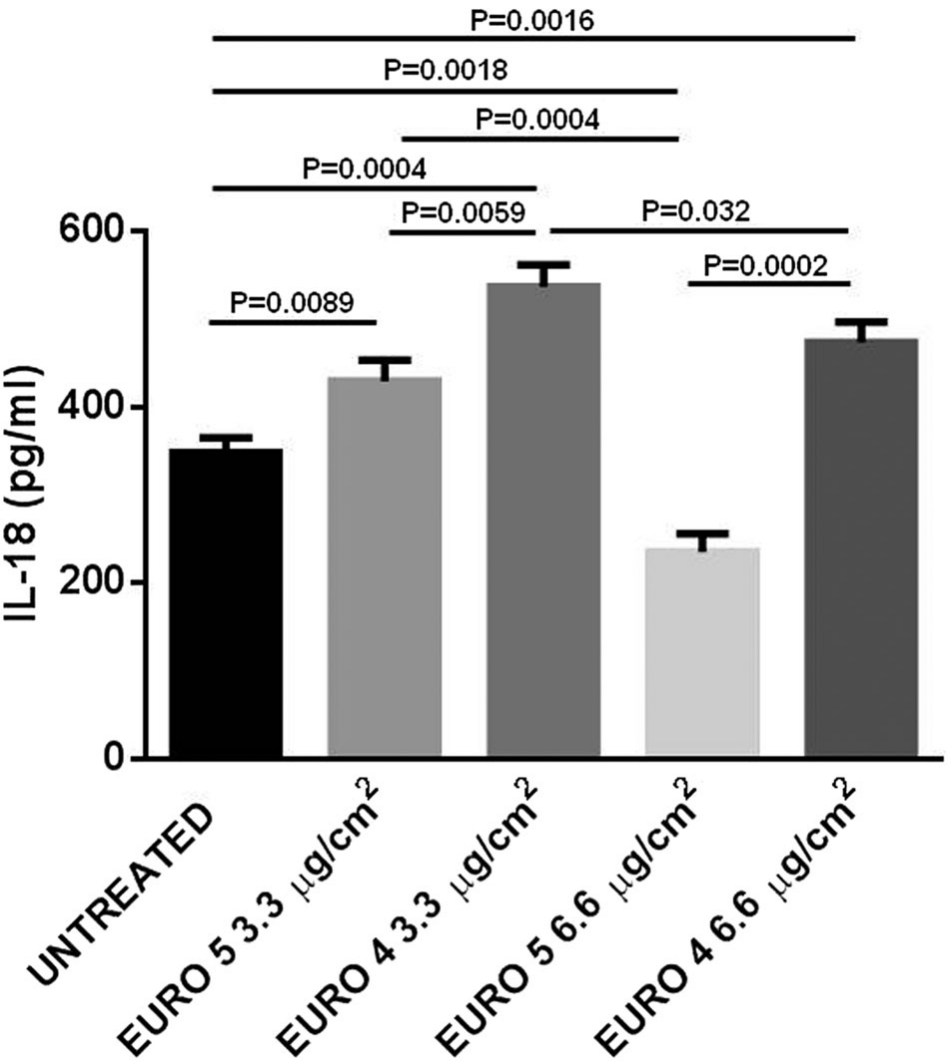
Effect of Euro 5 and Euro 4 DEPs, before any after-treatment, on IL-18 production evaluated in Normal Human Bronchial Epithelial (NHBE) cell supernatants. Cytokine release was analyzed using a commercially available ELISA kit. NHBE cells were stimulated for 24 h with Euro 5 and Euro 4 DEPs, before any after-treatment. After cell exposure to DEPs, supernatants were collected and analyzed. All the supernatants were run in quadruplicate in three different assays (*N* = 3) and the results are shown as mean ± SD. For 3.3 μg/cm^2^: *P* = 0.0089 Euro 5 *vs* untreated; *P* = 0.0004 Euro 4 *vs* untreated; *P* = 0.0059 Euro 5 *vs* Euro 4. For 6.6 μg/cm^2^: *P* = 0.0018 for Euro 5 *vs* untreated; *P* = 0.0016 for Euro 4 *vs* untreated; *P* = 0.0002 for Euro 5 *vs* Euro 4. For a dose-depending comparison: *P* = 0.0004 Euro 5 3.3 *vs* 6.6 μg/cm^2^; *P* = 0.032 Euro 4 3.3 *vs* 6.6 μg/cm^2^

All the particles considered for this study were able to induce a significant IL-18 production (Fig. 7), except for Euro 5 DEPs at 6.6 μg/cm^2^: at this concentration, a significant decrease of IL-18 production was observed. Respect to untreated samples, higher levels of IL-18 were detected in the NHBE cell culture supernatants at the DEP concentration of 3.3 μg/cm^2^, with an increase of IL-18 production due to Euro 4 > Euro 5.

As shown in Fig. 7, at the concentration of Euro 5 DEPs of 6.6 μg/cm^2^, a decrease of IL-18 level was observed, with respect to 3.3 μg/cm^2^. This effect could be ascribed to the higher percentage of necrotic cells observed in the same experimental condition (Fig. 4c), in which IL-18 production is higher in NHBE cells exposed to Euro 4, with respect to cells exposed to Euro 5 DEPs.

## Discussion

A proposed model for the pathogenesis of RA hypothesizes that RA-related autoimmunity can be generated in an extra-articular site. Several investigators have suggested that the lung may be involved in RA pathogenesis^21–24,26^. Moreover, it has been reported that protein citrullination in epithelial cells could be common ground for joints and pulmonary involvement^26,43–45^. In this  context, epithelial autophagy disregulation induced by stress agents, such as air pollution, may be an important immunological checkpoint for protein citrullination and, consequently, autoantibodies induction.

In this study, we have shown that NHBE cell exposure to Euro 4 and Euro 5 carbonaceous particles, the main component of particulate air pollution, can impair the normal function of bronchial epithelial cells, also affecting their ability to adapt to stress agents. Different studies focused on the changes in epithelial cell behaviour and function after the exposure to DEPs, but most of them have been carried out in adenocarcinoma human alveolar basal epithelial and in immortalized human bronchial epithelial cell lines^46^. It is well documented that DEPs internalized by human lung epithelial cells cause cell membrane alterations, cytoplasmic vacuolization, nuclear morphological modifications and increased pro-inflammatory cytokine release^47^. Nevertheless, few studies have been performed on the effects of air pollutants and, in particular, of DEPs on NHBE cells^48^. Our results are in accordance with the study of Matsuo and colleagues^7^, which demonstrated that DEPs were able to induce apoptosis and necrosis in NHBE cells but, in most of the previous studies, changes in autophagy and citrullination have not been yet investigated in these cells.

Although Euro 5 diesel engine emissions (in terms of mass in g/km) are lower than Euro 4, for certain aspects Euro 5 seemed to exert a more toxic effect than Euro 4^25^. In view of this, we examined the response of NHBE cells to Euro 4 and Euro 5 carbon particle exposure, in order to investigate their capacity to affect cell viability, induce autophagy, elicit protein citrullination and stimulate the release of the pro-inflammatory cytokine IL-18.

Our results showed that Euro 4 and Euro 5 carbon particles affected the viability, inducing a significant increase of apoptotic and necrotic cells and that Euro 4 particles were able to induce NHBE cell apoptosis and necrosis at a lower extent with respect to Euro 5, as previously observed by our group in skin cells^25^. As reported in the study of Mastrofrancesco and colleagues, we could hypothesize that the higher cytotoxic potential of Euro 5, with respect to Euro 4 particles, could be attributed to the slightly higher graphitic degree of Euro 5 DEPs, responsible for a different stabilization of radical species on DEP surface^25^. Anyway, more analyses are needed to confirm this hypothesis.

Moreover, cell exposure to Euro 4 and Euro 5 carbon particles was able to induce a significant increase of the autophagic flux, in terms of LC3-II level. p62 is localized to the autophagosome via LC3 interaction and is constantly degraded by the autophagy–lysosome system, so that change of autophagy could lead to modifications in p62 levels. Because the p62 level can also be influenced by other factors, independently of autophagy, the analysis of p62 marker alone would not have been accurate^49–51^. However, p62 level in NHBE cells seemed to be not significantly affected by the DEP exposure. We detected a higher increase in the autophagic flux when the process was stressed (as in the presence of specific lysosomal protease inhibitors), indicating that LC3-II accumulates when the lysosomal degradation is inhibited, and this would mean an enhancement of the autophagic flux^51^. Taking into account these results, we can conclude that DEPs stimulated autophagy, and this effect seemed to be more evident for Euro 5 DEPs.

To notice, we used low particle concentrations in our experimental conditions. DEP concentrations higher than 100 μg/ml have been used in other in vitro studies, in order to evaluate cell responses^52–54^. Li and colleagues have previously estimated that a biological relevant tissue culture concentration of DEPs ranges from 0.2 to 20 μg/cm^2^, so the DEP experimental concentrations applied in our work fall within this range^54^. As described by Kreyling and colleagues^55^, modern models are available to estimate realistic particle dosimetry in lungs, so the lack of this aspect could be considered a limit of the study. The aim of the present study was not to reflect exposure to particles in vivo, but rather to investigate the effect of DEPs in an in vitro model of bronchial epithelial cells, in particular monitoring cell viability and citrullination. This was a preliminary investigation for the comprehension of the potential relationship between autoimmune diseases (RA, in particular) and DEP exposure, so more studies are needed to confirm this speculation.

Interestingly, in our previous study performed in vitro on human lymphocytes from healthy subjects, the impact of DEPs on T lymphocytes phenotype and function was also pointed out. Exposure to Euro 4 or Euro 5 particles, at the same concentrations used in the present study, significantly suppressed IL-2 production in CD4^+^ and CD8^+^ T cells and a significant reduction for IFN-γ production, after DEP treatment, was observed with both compounds^38^. In this study, it was formerly hypothesized that the effects of DEP exposure on lymphocytes could lead to a defective immune surveillance, to an abnormal persistence of activated T cells and could promote the development or the progression of diseases, such as cancer and autoimmune diseases^38^.

We also observed a significant increase in protein citrullination, monitored by vimentin citrullination levels (since vimentin is known to be involved in citrullination due to inflammatory and/or disease-related conditions, such as in RA), and in PAD activity in NHBE cells exposed to both types of particles. These results, besides being in line with literature data on the cytotoxic effects of UFPs^46,47^, strengthen the tight correlation between autophagy and citrullination. It has been described that the presentation of citrullinated (and not the unmodified) peptides in several cell types is associated with autophagy, and that the presentation of citrullinated peptides can occur only after serum starvation, the typical autophagic stimulus^56^. These findings are also consistent with previous data, demonstrating the role of the autophagic process in the generation of citrullinated peptides. Human synoviocytes, treated with an autophagy inducer, revealed an increase of PAD activity, with consequent generation of citrullinated proteins, and showed the existence of a relationship between the autophagic response and the presence of anti-citrullinated protein antibodies (ACPA) in RA patients^57^. In this perspective, citrullination has to be considered the event, which plays a physiological role in the regulation of protein folding and in the degradation during cell death and tissue inflammation^58,59^ and that may lead to the development of adaptive responses specific for neo-epitopes exposed in modified self-proteins. The evidence linking autophagy to citrullination of antigens by APCs raises several intriguing points related to autoimmunity. External environmental stimuli may affect the local bronchial epithelial cells to induce autophagy and with it citrullination, creating a substrate for autoreactivity. Indeed, one of the strongest disease associations with citrullination is RA, so ACPA are used as a marker of the disease. Autophagy may be the common feature of several stress factors that may drive the adaptive responses to citrullinated self-proteins in RA autoimmunity^31^.

Finally, we demonstrated that NHBE cells exposed to both types of DEPs released IL-18, a key pro-inflammatory cytokine that works as a pleiotropic immune regulator by IFN-γ production, in the culture medium. Of note, IL-18 has been reported to play a potential pathological role in RA and SLE^60–64^.

We observed that Euro 4 carbon particles were able to induce a significant IL-18 production by NHBE cells both at the lower (3.3 μg/cm^2^) and the higher (6.6 μg/cm^2^) concentration. Euro 5 particles stimulated IL-18 release only at the lower concentration (3.3 μg/cm^2^), while a significant decrease of IL-18 production was observed when the cells were challenged with Euro 5 particles at the higher concentration (3.3 μg/cm^2^), maybe due to the higher percentage of late apoptotic/necrotic cells in this experimental condition (Fig. 7 and Fig. 4c).

In conclusion, the current findings demonstrate that DEP carbon core affects NHBE cell viability, enhancing apoptosis and necrosis, increases the autophagic flux, induces protein citrullination and IL-18 secretion, and stimulates PAD activity in NHBE cells. Taken together, all these data suggest that chronic exposure to environmental DEPs could be responsible for the generation of autoimmune RA features in genetically susceptible individuals^26^.

## Electronic supplementary material

Supplementary Figure 1

Supplementary Figure 2

Supplementary Figure 3

Supplementary Figure Legends
