# Supplementary figures and images for: Diesel exhaust particles induce autophagy and citrullination in Normal Human Bronchial Epithelial cells
Source: Cell Death Dis. 2018 Oct 19;9(11):1073. doi: 10.1038/s41419-018-1111-y (PMC6195610; doi:10.1038/s41419-018-1111-y)

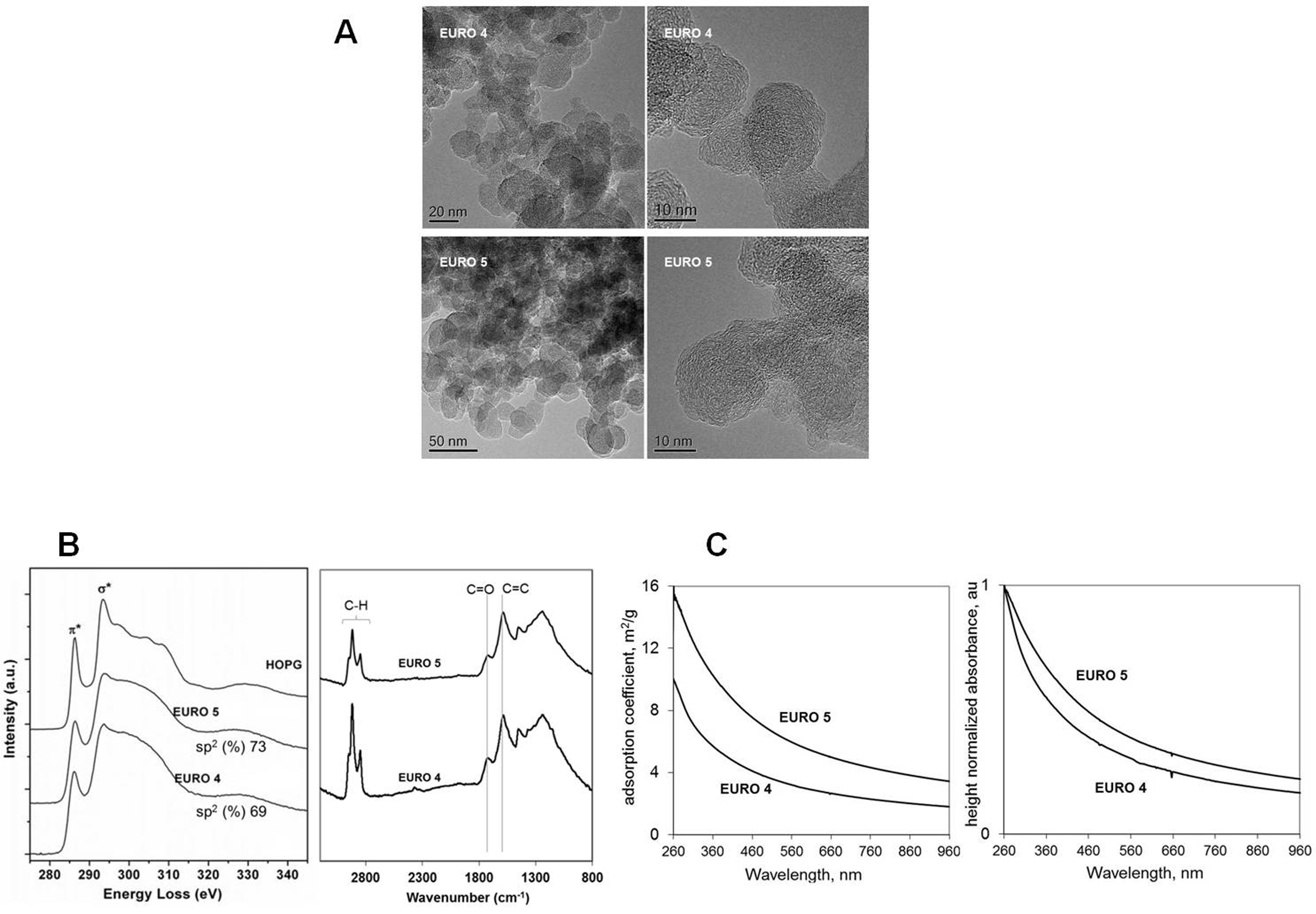

Supplement: Supplementary file 1 — Supplementary Figure 1 [file 41419_2018_1111_MOESM1_ESM.tif]

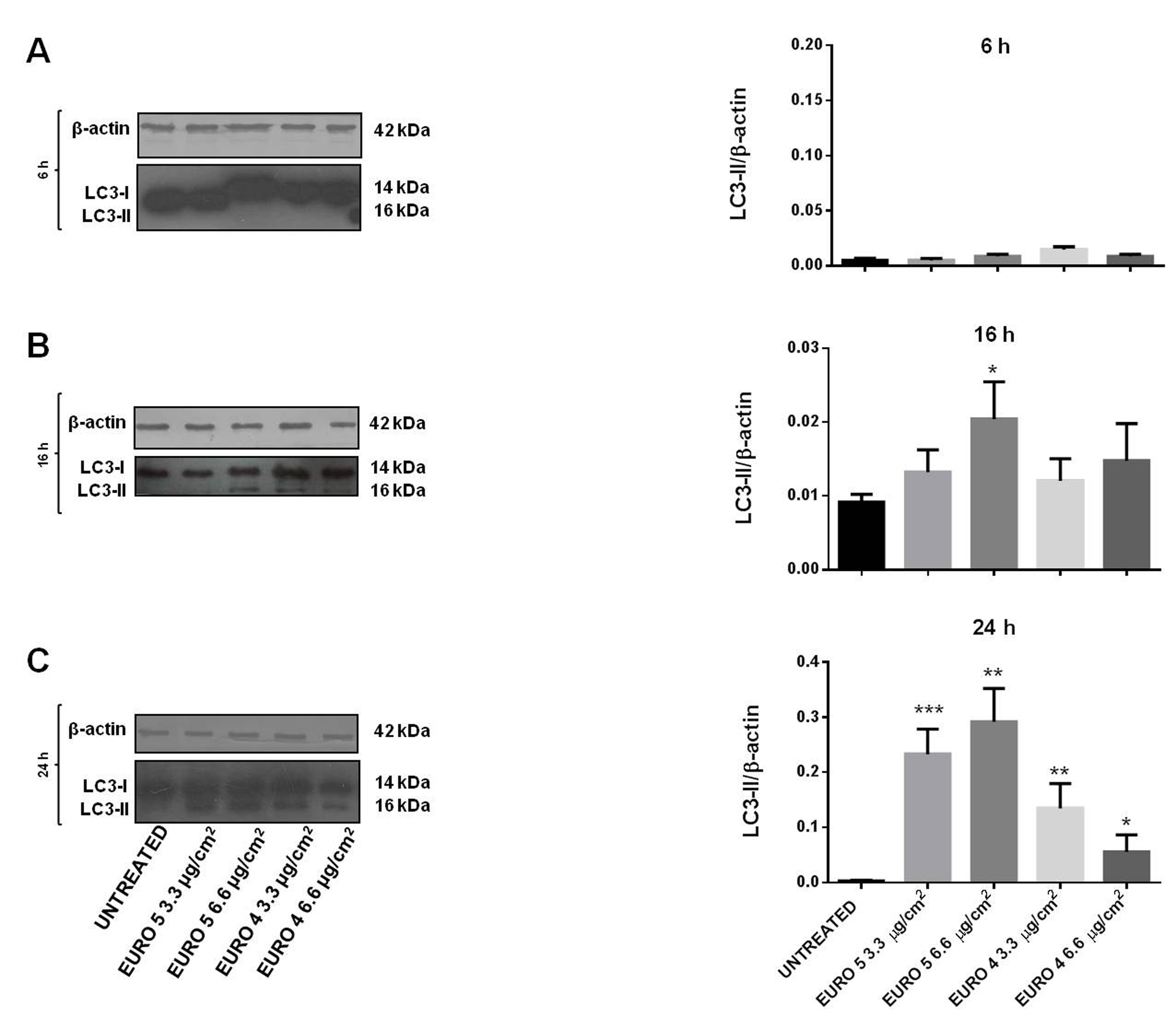

Supplement: Supplementary file 2 — Supplementary Figure 2 [file 41419_2018_1111_MOESM2_ESM.tif]

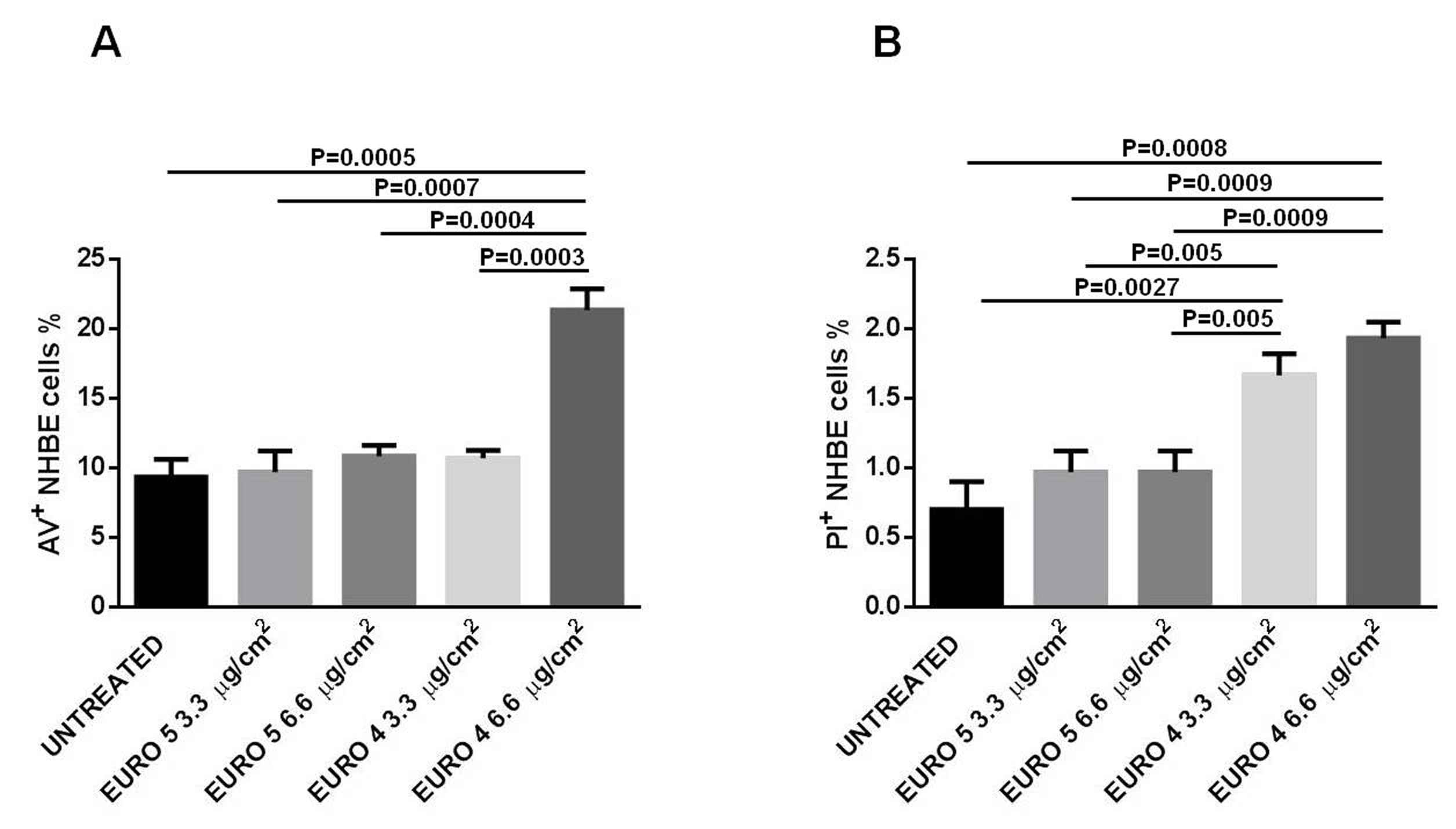

Supplement: Supplementary file 3 — Supplementary Figure 3 [file 41419_2018_1111_MOESM3_ESM.tif]
