# Supplementary material for: Diesel exhaust particles induce autophagy and citrullination in Normal Human Bronchial Epithelial cells
Source: Cell Death Dis. 2018 Oct 19;9(11):1073. doi: 10.1038/s41419-018-1111-y (PMC6195610; doi:10.1038/s41419-018-1111-y)
Supplement: Supplementary file 4 — Supplementary Figure Legends [file 41419_2018_1111_MOESM4_ESM.doc]

**SUPPLEMENTARY FIGURE LEGENDS**

**Supplementary Fig. 1. Chemico-physical characteristics of Euro 4 and Euro 5 carbon particles, before any after-treatment. a** Transmission electron microscopy (TEM) of Euro 4 and Euro 5 particulate shows a similar micro- and nano-texture, consisting of irregularly shaped compact aggregates of almost spherical primary particles (diameter 10-20 nm, left panels). Short graphene layers are clearly discernible in the High-Resolution TEM (HRTEM) images (right panels) and arranged in a typical turbostratic fashion. The particle surface appears irregular, indicating the presence of sp^3^-hybridized carbons defects in the carbonaceous network. **b** Electron Energy Loss Spectroscopy (EELS) - attenuated total reflection infrared (ATR-IR) spectroscopy. The Energy-Loss Near Edge Structure (ELNES) of the carbon- K-ionization edge deduced from the EELS spectra are here reported. Carbon-K-ionization edges (left panel) and ATR-IR spectra (800-3200 cm^-1^, right panel) of Euro 4 and Euro 5 carbon particles. The spectrum of highly ordered pyrolytic graphite is also displayed for comparison and corresponds to a graphitization degree, expressed in terms of sp^2^ % of 100. The peak located at 285 eV (labeled π*) in left image arises from transitions to the unoccupied antibonding π*-states, while the intense peak at higher energy losses (>292 eV) is due to transitions to antibonding π*-states. The peak located around 285 eV is usually referred as “graphitic peak” and it is indicative of aromatically bound carbons, while the peak at 292 eV corresponds to the π peak of the C=C bond resonance. The presence of both signals is typical of carbonaceous conjugate networks, as testified by the graphite spectrum. The predominant binding properties are similar in Euro 4 and Euro 5 DEPs and agree with EELS data reported for other diesel particulate samples. The graphitization degree is slightly more pronounced in the Euro 5 diesel exhaust particles (DEPs; 73% *vs* 69%), indicating a lower presence of defective sites compared to Euro 4 DEPs. The spectra appear quite similar and no significant differences are detectable by ATR-IR inspection in both operating conditions (right panel). The broad shape of the spectra is typical of a complex carbon network. Bands originated from the vibrations of oxygenated functionalities (1650-1750 cm^-1^, C=O stretching vibrations from carbonyl and carboxylic groups) and graphitic domains (1500-1600 cm^-1^, skeletal vibration of the sp^2^ moieties) are the most characteristic features of both DEP spectra. The broad band in the 1300-1100 cm^-1^ region is ascribable to the overlapping of C-OH and C-O stretching vibrations. Weak and broad signals in the 2950-2850 cm^-1^ region, attributable to aliphatic groups (mainly methylene) are also detected, probably residuals of unburned or partially burned fuel, not completely removed by solvent extraction. **c** UV spectra of Euro 4 and Euro 5 carbon particles. The DEP spectral features and hydrodynamic diameter were evaluated in N-methyl pyrrolidinone (NMP) suspensions. The height normalized UV-Vis spectra of DEPs exhibited a broad shape degrading from UV toward visible region typical of complex carbon-based materials produced in combustion processes and ascribable to the presence of highly conjugated systems. The spectral shape appeared quite similar for both samples but the specific absorptions (sensitive to sp^2^/sp^3^ ratio) were quite different, both in the UV (300 nm) and in the visible (500 nm) regions. In particular, the specific absorption values are higher for Euro 5 than Euro 4 DEPs (13 and 7.5 m^2^/g at 300 nm and 6.8 and 3.7 m^2^/g at 500 nm for Euro 5 and Euro 4, respectively), indicating that the graphitization degree is slightly more pronounced in the Euro 5 DEPs, in accordance with EELS data. Overall, the specific absorption values are similar to those of carbons with a high graphitization degree and a good level of structuration (furnace carbon blacks, mature soot from benzene laminar flame).

**Supplementary Fig. 2. Time–response analysis of LC3-II levels in Normal Human Bronchial Epithelial (NHBE) cells after Euro 5 and Euro 4 carbon particle exposure.** LC3-II Western blot analysis of NHBE cells lysates after treatment with Euro 5 and Euro 4 DEPs at 3.3 and 6.6 μg/cm^2^ at different times (6, 16 and 24 h). These preliminary experiments were performed to monitor the time-response of LC3-II in NHBE cells. Statistically significant differences *versus* untreated are reported. **a** Western blot analysis of LC3-II in lysates from NHBE cells after treatment with Euro 5 or Euro 4 carbon particles for 6 h (left panel). Densitometry analysis of specific protein levels relative to β-actin is shown (right panel). Values are expressed as mean ± SD obtained from three experiments performed in NHBE cells (N=3). **b** LC3-II Western blot analysis of NHBE cell lysates after treatment with Euro 5 or Euro 4 particles for 16 h (left panel). Densitometry analysis of LC3-II levels relative to β-actin is also shown (right panel). Values are expressed as mean ± SD obtained from three experiments performed in NHBE cells (N=3). *P=0.0196 for Euro 5 6.6 μg/cm^2^ *vs* untreated. **c** LC3-II Western blot analysis of NHBE cell lysates after treatment with Euro 5 or Euro 4 particles for 24 h (left panel). Densitometry analysis of LC3-II levels relative to β-actin is also shown (right panel). Values are expressed as mean ± SD obtained from three experiments performed in NHBE cells (N=3). For 3.3 μg/cm^2^: ***P=0.0009 for Euro 5 *vs* untreated; **P=0.0073 for Euro 4 *vs* untreated. For 6.6 μg/cm^2^: **P=0.0011 for Euro 5 *vs* untreated; *P=0.0423 for Euro 4 *vs* untreated.

**Supplementary Fig. 3. Effect of Euro 5 and Euro 4 carbon particles on cell viability: evaluation of apoptosis and necrosis in Normal Human Bronchial Epithelial (NHBE) cells at 48 h.** Analysis of apoptosis and necrosis by flow cytometry in NHBE cells untreated and treated for 48 h with Euro 4 and Euro 5 carbon particles. Results shown are representative of three experiments performed in NHBE cells(N=3). **a** Apoptosis levels in NHBE cells exposed to DEPs are reported as the percentage of AV^+^ cells. For 3.3 μg/cm^2^: P*=*0.785 for Euro 5 *vs* untreated; P*=*0.1706 for Euro 4 *vs* untreated; P=0.3486 for Euro 5 *vs* Euro 4. For 6.6 μg/cm^2^: P=0.1523 for Euro 5 *vs* untreated; P*=*0.0005 for Euro 4 *vs* untreated; P=0.0004 for Euro 5 *vs* Euro 4. For a dose-depending comparison: P=0.3022 for Euro 5 3.3 *vs* 6.6 μg/cm^2^; P=0.0003 for Euro 4 3.3 *vs* 6.6 μg/cm^2^. P=0.0007 for Euro 5 3.3 *vs* Euro 4 6.6 μg/cm^2^; P=0.778 for Euro 5 6.6 *vs* Euro 4 3.3 μg/cm^2^. **c** Necrosis levels in NHBE cells exposed to DEPs are reported as the percentage of PI^+^ cells. For 3.3 μg/cm^2^: P=0. 1404 for Euro 5 *vs* untreated; P=0.0027 for Euro 4 *vs* untreated; P=0.005 for Euro 5 *vs* Euro 4. For 6.6 μg/cm^2^: P=0.1404 for Euro 5 *vs* untreated; P=0.0008 for Euro 4 *vs* untreated; P=0.0009 for Euro 5 *vs* Euro 4. For a dose-depending comparison: P>0.999 for Euro 5 3.3 *vs* 6.6 μg/cm^2^; P=0.0734 for Euro 4 3.3 *vs* 6.6 μg/cm^2^. P=0.0009 for Euro 5 3.3 *vs* Euro 4 6.6 μg/cm^2^; P=0.005 for Euro 5 6.6 *vs* Euro 4 3.3 μg/cm^2^.
